# Clinical Value and Time Course of Pericoronary Fat Inflammation in Patients with Angiographically Nonobstructive Coronaries: A Preliminary Report

**DOI:** 10.3390/jcm10081786

**Published:** 2021-04-20

**Authors:** Valeria Pergola, Marco Previtero, Annagrazia Cecere, Vittorio Storer, Teresa Castiello, Anna Baritussio, Giulio Cabrelle, Donato Mele, Raffaella Motta, Alida Patrizia Caforio, Sabino Iliceto, Martina Perazzolo Marra

**Affiliations:** 1 Department of Cardiac, Vascular, Thoracic Sciences and Public Health, University of Padua, 35128 Padua, Italy; marco.previtero@aopd.veneto.it (M.P.); annagrazia.cecere@unipd.it (A.C.); vittorio.storer@studenti.unipd.it (V.S.); anna.baritussio@aopd.veneto.it (A.B.); DONATO.MELE@UNIPD.IT (D.M.); alida.caforio@unipd.it (A.P.C.); sabino.iliceto@unipd.it (S.I.); martina.perazzolomarra@unipd.it (M.P.M.); 2 Department of Cardiology Croydon Health Service, London CR7 7YE, UK; teresa.castiello@nhs.net; 3 Department of Medicine, Institute of Radiology, University of Padua, 35128 Padua, Italy; giulio.cabrelle@aopd.veneto.it (G.C.); RAFFAELLA.MOTTA@UNIPD.IT (R.M.)

**Keywords:** pericoronary fat inflammation, cardiac computed tomography, MINOCA, takotsubo, myocarditis

## Abstract

The introduction of high-sensitivity cardiac troponin allowed identifying a proportion of subjects with chest pain and electrocardiographic changes suggestive of myocardial infarction showing <50% coronary artery stenosis. PFAI is a coronary CT marker proved to predict outcome in ischemic heart disease. Based on CMR findings, patients were divided into myocarditis (*n* = 15), MINOCA (*n* = 14) and TTS (*n* = 9) groups. The aim was to estimate the value of pFAI in these groups compared to 12 controls. To evaluate the coronary inflammation “time course,” 20 patients underwent CMR and coronary CT scan within 8 days from the onset, the others within 60 days. There were higher values of pFAI in myocarditis (−86.45 HU), MINOCA (−84.63 HU) and TTS (−84.79 HU) compared to controls (−96.02 HU; *p* = 0.0077). Among patients who underwent CT within 8 days from onset, the MINOCA had a significantly higher pFAI value (−76.91 HU) compared to the control group (−96.02 HU; *p* = 0.0001). In the group that underwent CT later than 8 days, elevated pFAI values persisted only in the myocarditis and TTS groups, and there was no difference between MINOCA and controls. Our study shows that in patients with a diagnosis of MINOCA, there is acute coronary inflammation, which is more evident within one week from the acute event but tends to disappear with time.

## 1. Introduction

The recent introduction of high-sensitivity cardiac troponin has allowed identifying an increasing number of subjects with typical chest pain and electrocardiographic changes suggestive of acute myocardial infarction (AMI), although coronary angiograms

Demonstrate less than 50% of coronary stenosis. The third universal definition of AMI classified AMI into five types, the first based on the rupture of plaques and the second on other ischemic imbalances (e.g., endothelial dysfunction, spasm, embolisms, etc.). The role of inflammation in the pathogenesis of these types is currently under debate [1].

Cardiac magnetic resonance (CMR) enables identifying whether myocardial injury represents underlying myocarditis, takotsubo syndrome (TTS) or true myocardial infarction with nonobstructive coronary arteries (MINOCAs) [2].

The evaluation of coronary inflammation by coronary computed tomography (CCT) in these subsets of patients remains unknown. Epicardial CAs are covered by adipose tissue. Recently, pFAI measured from CCT was able to detect biopsy-result-proven vascular inflammation in patients undergoing cardiac surgery [3].

The perivascular fat attenuation index (pFAI) is a new imaging marker of inflammation using a CCT scan that has been proven to predict the outcome of subjects with ischemic heart disease. A cut-off of −70.1 Hounsfield units (HUs) was identified as an indicator of increased risk of cardiovascular death [4].

The aim of the present study is to evaluate the presence and extent of coronary inflammation in a court of patients with myocardial damage and no angiographically significant coronary atherosclerosis (myocarditis, MINOCA and TTS) and compare the findings with healthy subjects. Additional aims are to evaluate the time course of coronary inflammation on CCT and evaluate the correlations between coronary inflammation and CMR-derived myocardial strain (longitudinal, radial and circumferential strain).

## 2. Materials and Methods

From the CMR register in our hospital (Padua University), we retrospectively reviewed 498 patients who underwent the procedure from April 2016 to January 2020 for suspected myocarditis, MINOCA or TTS.

We included all patients with nonobstructive coronary plaques who underwent CCT between 1 and 60 days from the initial diagnosis. Exclusion criteria were: presence of at least moderate coronary lesions; low rise in myocardial cytolysis indices; atrial fibrillation; presence of chronic infectious diseases or other inflammatory conditions such as active neoplasm, asthma, eczema, allergy, rheumatoid arthritis, systemic lupus erythematosus, Crohn’s disease, ulcerative colitis or any concurrent physical illness that in the judgment of investigators was a potential confounder to the hypothesis (e.g., concurrent hypertrophic or noncompaction cardiomyopathy, moderate to severe left ventricular hypertrophy of any other cause).

We finally selected 38 patients. Of these cases, 15 patients had a confirmed diagnosis of myocarditis (according to the 2013 ESC position statement) [5], 14 patients of MINOCA (no identifiable clinically overt cause for the acute MI event) [6] and 9 of TTS (according to the Heart Failure Association of the European Society of Cardiology diagnostic criteria for TTS) [7]. All patients underwent CCT only once.

Data were compared with 12 patients who underwent a CCT scan because of a workup of atypical chest pain. All control subjects had no more than 1 risk factor for CA disease and no more than mild coronary atherosclerosis (CAD-RADS = 0 or 1).

For each MINOCA patient, the infarct-related culprit artery was assigned on the basis of the late gadolinium enhancement (LGE) location shown by the CMR images or, if LGE was not present, using acute wall motion abnormalities on CMR or echocardiography. The association between CA distribution and myocardial segments was made on the basis of the American Heart Association recommendations [8].

In TTS cases, echocardiograms were performed 1 to 3 months after the event to confirm the temporary nature of the acute wall motion abnormalities.

Echocardiographic examinations were performed using Philips iE33 (iE33 xMATRIX Philips Healthcare, Best, the Netherlands), GE Vivid7 or GE Vivid9 (GE; General Electric, Milwaukee, WI, USA) ultrasound machines. Three cardiac cycles in each of the standard parasternal long-axis, short-axis and apical four-, three- and two-chamber views were obtained at a frame rate of at least 40. All images were exported as DICOM files for vendor-independent software (TomTec 2D Cardiac Performance Analysis (2DCPA) version 1.2.1.2) and analyzed by an experienced cardiologist.

CCT was performed with an MDCT 320-slice scanner (Aquilion ONE, Canon Medical Systems, Tustin, CA, USA). CCT protocol scanning with prospective ECG gating was performed during breath hold, using 320 slices with a collimated slice thickness of 0.5 mm. Patients were infused with 50 to 70 nonionic contrast agents (iomeprol: 816.50 mg/mL, Iomeron 400, Bracco Imaging, Milan, Italy). Furthermore, to reach less than 65 bpm, metoprolol succinate (5–40 mg) was administered intravenously unless contraindicated. Five milligrams of sublingual nitroglycerin was also administered before the examinations to all patients.

Images were acquired after iodinated contrast administration using the “bolus tracking” technique and reconstructed in post-processing by the volume rendering technique and curved multiplanar reformations with vessel centerline analysis.

**Figure 1:**
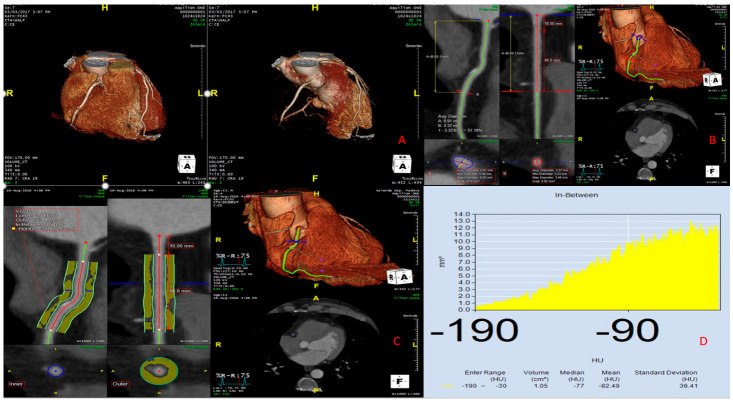
Example of how to measure the pericoronary adipose tissue. (**A**) Three-dimensional (3D) coronary CT reconstruction obtained using Aquarius TeraRecon. (**B**) Selection of the right coronary artery (RCA) and its region of interest using Aquarius TeraRecon. (**C**) Pericoronary tissue analysis showing the distribution of values between −190 and −30 HU. (**D**) Histogram showing the distribution of pericoronary tissue values in the range of −190 and −30 HU.

PFAI was assessed using Aquarius Workstation version 4.4.13.P4 (TeraRecon Inc., Foster City, CA, USA). As previously reported, perivascular fat was defined as the adipose tissue within a radial distance from the outer vessel wall equal to the diameter of the vessel. We traced the proximal 40 mm segments starting 10 mm from the right coronary artery (RCA) ostium. In agreement with previous studies [3], pFAI was ascertained by quantifying the weighted perivascular fat attenuation based on the attenuation histogram of perivascular fat within the range of −190 to −30 HU. We measured pFAI in all three major coronary arteries but restricted the analysis to the RCA (Figure 1) [4].

Values were compared to those of 12 healthy subjects. All measurements were independently made by two experienced observers to assess interobserver agreement.

CMR was performed with a 1.5 T scanner (Magnetom Avanto, Siemens Healthcare, Munich, Germany) with a dedicated protocol, including spin-echo and postinjection sequence contrast. The images were then evaluated with a macOS analysis program (Cvi42 software, Circle Cardiovascular Imaging Inc.; Calgary, AB, Canada).

Biventricular morphological and functional evaluation was performed on a series of kinetic images acquired by steady-state free precession (SSFP) in sequential short-axis sections from the mitral valve to the cardiac apex (section thickness, 6 mm; gap, 0 mm; repetition time from 2.5 to 3.8; echo time from 1.1 to 1.6; average spatial resolution, 1.5 × 2.4 mm; flip angle from 45 to 60°; time resolution from 30 to 40 ms) and in long-axis projections (in 2–4 rooms).

The right ventricle (RV) was evaluated with transaxially balanced kinetic images from the outflow tract to the diaphragm in a two-chamber projection.

The ventricular volumes were indexed by the body surface area.

T1-weighted turbo spin-echo images, obtained in the axial planes, were evaluated to determine the presence of myocardial adipose infiltration, defined as a clear interruption of the dividing line between gray myocardium and white epicardial fat. In some cases, T2-weighted short tau inversion recovery (STIR) sequences were used to confirm the presence of intramyocardial fat.

Gradient echo or inversion recovery sequences were used to evaluate the presence of LGE, performed 10–15 min after the injection of 0.2 mmol/kg of gadolinium (Gadobenate Dimeglumine, Multihance, Bracco) and evaluated on the same sections of the cine images. The inversion time was set manually to optimize image quality and ensure maximum contrast due to the suppression of normal myocardium. Tissue characterization by CMR was conducted using Cvi42 software. The presence or absence of myocardial edema was quantified and coded dichotomously (with a signal intensity of >2SD compared with that of the skeletal muscle) [9]. For the presence of LGE, a cut-off of 5SD was used [10]

Presence, pattern and localization of LGE were judged by observers unaware of the patients’ clinical data using axial, long-axis and short-axis sections.

**Figure 2:**
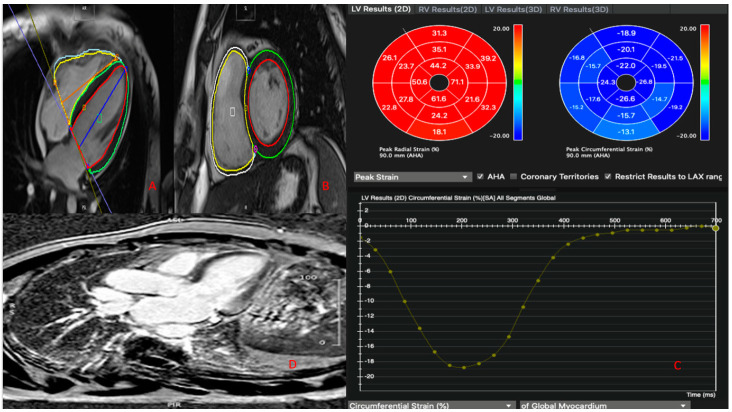
(**A**) Apical four-chamber CMR view. (**B**) Short-axis CMR view. The red and yellow lines trace the telediastolic endocardial border of the left and right ventricle, respectively. The green and white lines trace the telediastolic epicardial border of the left and right ventricle, respectively. (**C**) Two-dimensional LV radial and circumferential strain. The bullseyes show the average radial (red bullseye) or circumferential (blue bullseye) values for each of the 16 segments of the myocardium. The bottom of the figure shows the myocardial deformation curve. (**D**) Long-axis CMR view showing LGE.

Myocardial strain was analyzed with dedicated software (Cvi42) using the same kinetic images of cardiac magnetic resonance used for three-chamber, short-axis and long-axis morphofunctional evaluation (Figure 2).

Global longitudinal strain, global radial strain and global circumferential strain were analyzed for both ventricles.

The study was conducted according to the guidelines of the Declaration of Helsinki. Informed consent was obtained from all subjects involved in the study.

## 3. Statistical Analysis

Statistical analyses were performed with SPSS 26 software for macOS (SPSS Inc., Chicago, IL, USA). Continuous variables were reported as the mean and standard deviation, and categorical variables were reported as frequencies and percentages.

Comparison between categorical variables was performed by a Chi-square test or Fisher’s exact test if appropriate.

Continuous variables were analyzed according to the Shapiro–Wilk test to verify if the distribution was normal; if the distribution was normal, the Student’s *t*-test was used, and if the distribution was not normal, the Mann–Whitney test was used.

In cases where, for the same continuous variable, several groups were analyzed, the Kruskal–Wallis test was used.

In cases where the correlation between two continuous variables was analyzed, Pearson’s test was used.

Intraclass correlation coefficients (ICCs) were calculated using the two-way mixed-effects model. The repeatability coefficient was defined as 1.96 × the standard deviation of the absolute differences of each measure.

A statistical significance of *p* < 0.05 was assumed for all tests.

## 4. Results

The study population included 38 patients with a mean age of 43.79 ± 16.50 years. Of these 15 subjects (39%), 16 men (42%) had a discharge diagnosis of myocarditis, 14 patients of MINOCA (37%) and 9 (24%) of TTS.

Cardiovascular risk factors, presenting symptoms, ECG, laboratory and echocardiographic data are summarized in Table 1. There was no statistically significant difference between patients and normal controls in terms of risk factors, except for stressful events, which were more frequent in the TTS group.

The mean CRP value was 22.82 ± 53.77 mg/L, and the mean TnI HS value was found to be 8446.96 ± 13,515 ng/L. As expected, no normal subjects had elevated CRP or TnI HS. These laboratory findings were evaluated at the time of the acute event.

On echocardiographic examination, all controls showed normal diastolic diameters and normal systolic function with no evidence of regional wall motion abnormalities. Changes in regional kinetics were evident in 21 patients (55%), and pericardial effusion was present in 2 patients (6%).

On CMR imaging. 22 patients (58%) had left ventricular edema in precontrast T2-weighted images, 27 patients (71%) had evidence of LGE in the left ventricle and 11 patients (29%) had an ischemic pattern (Table 2).

Left ventricle edema was observed in six patients with TTS (67%), in nine patients (60%) with myocarditis and in nine patients (50%) with MINOCA (*p* = NS).

LGE was present in 13 patients (87%) with myocarditis (epicardial or midwall enhancement), in 11 out of 14 patients with MINOCA (79%) (transmural or subendocardial enhancement) and in 3 patients with TTS (33%). In the remaining three patients with a confirmed diagnosis of MINOCA secondary to vasospasm, no LGE was detected.

LGE with an ischemic pattern was not observed in patients with myocarditis. RV edema and/or LGE were not present in any patient.

Details about LV and RV volumes and function are shown in Table 3.

There was no correlation between pFAI values and MRI-derived strain.

The CCT showed that nine patients (24%) were free from atherosclerotic lesions, six patients (16%) had trivial or mild lesions in the anterior descending coronary artery (LAD), six patients (16%) in the circumflex artery (LCX) and five patients (13%) in the of RCA. High-risk coronary plaques (defined as presenting at least one plaque showing at least one among the following: positive remodeling, napkin-ring sign, spotty calcifications or low-attenuation plaque) were significantly higher in unhealthy cases (48% of detected plaques) compared with controls (23% of detected plaques) (*p* = 0.003). Intraobserver intraclass correlation coefficients (ICCs) for PCAT attenuation were excellent (0.999).

The pFAI values in healthy patients were significantly lower than in patients with myocarditis, MINOCA and TTS. The pFAI mean value was −85.51 HU in the whole population and −96.02 in healthy controls (*p* = 0.0077).

In patients with myocarditis, the mean value was −86.45 HU, and the difference was statistically significant compared to healthy subjects. When we divided patients between early and late CT, we found the early group had a lower mean value of −85.35 HU, which was statistically significant compared to healthy subjects. The late group also presented a mean pFAI of −87.42 HU, which was statistically significant compared to normal subjects.

In patients with MINOCA, mean pFAI values were −84.63 HU, significantly higher compared to normal subjects (*p* = 0.0160). When we divided patients between early and late CT, we found the early group had a lower mean value of –76.91 HU, and the late group presented a mean pFAI of 96.99 HU, which was not statistically significant compared to normal subjects.

In patients with TTS, the mean value was −84.79 HU, which was statistically significant compared to the normal population. Separately analyzing early and late CT patients, we found higher mean values of –83.54 and −86.02 HU, respectively, which were statistically significant compared to healthy subjects. Details are shown in Table 4.

Patients with myocarditis and MINOCA showed a higher readmission rate compared to TTS and healthy subjects in the one-year follow-up (see details in Table 5). In our population, there was no correlation between pFAI values and follow-up data (*p* = 0.21; R = 0.224).

## 5. Discussion

In the present study, the attenuation index of pericoronary adipose tissue was used to compare patients diagnosed with myocarditis, MINOCA and TTS with healthy subjects.

Oikonomou et al. [4] proposed −70.1 HU as the cut-off value to determine high levels of pFAI. Our analysis showed that healthy subjects had a value of −96.27 HU, and all patients had lower values, with an average of −85 to −87 HU. Indeed, Oikonomou [4] included many patients with moderate (50–69%) and severe (≥70%) stenosis. Conversely, in our study, we only included patients with <50% CA stenosis. It is therefore evident that the presence of >50% stenosis can affect the density values of the pericoronary tissue, leading to an increase in tissue inflammation [3,11].

In our study, the difference between patients and healthy controls is evident, with the latter having significantly lower pericoronary tissue density values. This evidence is higher in the early group and showed a downtrend in the CCT performed after eight days from the onset of symptoms. This trend, although not statistically significant in patients with myocarditis and TTS, is significant in the MINOCA group, where the late CCT did not show a statistically significant difference in pFAI compared to control subjects. These data are in agreement with Gaibazzi et al. [12], who found higher mean pFAI values in patients with MINOCA/TTS compared with controls. In addition, our study demonstrated that inflammation is more pronounced in the acute phase and tends to ease off rapidly in MINOCA patients.

From these data, it is evident that inflammation of the pericoronary tissue plays an acute role in MINOCA compared to myocarditis and TTS, as demonstrated by the normalization of mean pFAI values in late CCT.

It was demonstrated that coronary vasospasm, a significant cause of type 2 AMI [1] and one of the possible underlying causes of MINOCA, is strictly associated with coronary inflammation [13], and our findings for the first time are in agreement with this hypothesis.

We did not demonstrate a correlation between pFAI values and prognosis; however, these data should be interpreted with caution, as our sample size is small, the follow-up is relatively short and there are too many possible covariates that may influence this correlation.

The clinical use of pFAI could therefore be useful in patients with an increase in myocardial cytolysis indices and angiographically nonobstructive CA to better understand the underlying mechanism of the diseases. Further studies with a bigger sample size are needed to verify the usefulness of pFAIin in predicting patients’ prognosis, as previous studies showed that patients with high levels of coronary inflammation have an increased risk of cardiac death [14].

## 6. Conclusions

In conclusion, the pFAI values were significantly lower in healthy controls compared to patients with myocarditis, MINOCA and TTS. Our study showed for the first time that patients with a diagnosis of MINOCA have acute coronary inflammation, which is more evident within one week from the acute event but tends to disappear with time. On the contrary, in patients with TTS and myocarditis, pFAI values are altered for a longer time.

Our data demonstrate that routine pFAI analysis through CCT in patients with elevated troponin, ECG abnormalities and nonobstructive coronary arteries could be a useful tool with pathophysiologic insights regarding the second type of AMI, the incidence of which is increasing. Larger studies are needed to identify its role in the prognosis of this subset of patients.

## 7. Study Limitations

The present study is limited by the small number of patients due to the narrow inclusion criteria and that it is a single-center study.

Another limitation is the number of exams performed after 8 days from the acute event, allowing potential recovery of myocardial edema on CMR.

Another limitation is related to the contraindications of both imaging procedures, such as claustrophobia, allergies to contrast agents, chronic renal failure and the exclusion of patients with atrial fibrillation.

## Figures and Tables

**Table 1:** Clinical and echocardiographic characteristics.

Variable	Myocarditis (*n* = 15)	MINOCA (*n* = 14)	Takotsubo Cardiomyopathy (*n* = 9)	All Patients (*n* = 38)	Healthy Controls (*n* = 12)	*p*-Value *
Age (years)	33.73 ± 13.40	50.00 ± 14.87	56.60 ± 13.37	43.79 ± 16.50	48.5 ± 10.93	0.713
Sex ratio (M/F)	4/11	10/4	2/9	16/22	4/8	0.279
Smokers	8 (53.3%)	6 (42.9%)	2 (22.2%)	16 (42.1%)	5 (41.6%)	0.979
Hypertension	1 (6.67%)	4 (28.6%)	2 (22.2%)	7 (18.4%)	3 (25%)	0.628
Diabetes mellitus	0 (0.0%)	0 (0.0%)	0 (0.0%)	0 (0.0%)	0 (0.0%)	1.000
Dyslipidemia	2 (13.3%)	2 (14.3%)	2 (22.2%)	6 (15.7%)	2 (16.7%)	0.124
Family history of CAD	4 (26.7%)	4 (28.6%)	4 (44.4%)	12 (31.6%)	3 (25%)	0.672
Fever	6 (40.0%)	2 (14.3%)	2 (40.0%)	10 (29.4%)	0 (0.0%)	0.041
Angina	2 (13.3%)	3 (21.4%)	1 (20.0%)	6 (17.6%)	0 (0.0%)	0.124
Previous thromboembolism	0 (0.0%)	1 (7.1%)	0 (0.0%)	1 (2.6%)	0 (0.0%)	0.558
Stressful event	0 (0.0%)	3 (21.4%)	9 (100.0%)	12 (31.6%)	0 (0.0%)	<0.001
Drug abuse	2 (13.3%)	0 (0.0%)	0 (0.0%)	2 (5.3%)	0 (0.0%)	0.402
Days between RM and CT	10.8 ± 8.74	14.07 ± 14.82	17.00 ± 14.66	12.88 ± 12.13	NA	NA
**Presentation**						
Chest pain	13 (86.7%)	12 (85.7%)	4 (44.4%)	29 (76.3%)	9 (75 %)	0.779
Dyspnea	0 (0.0%)	1 (7.1%)	0 (0.0%)	1 (2.6%)	1 (1%)	0.558
Palpitations	1 (6.67%)	0 (0.0%)	1 (11.1%)	2 (5.2%)	2 (2%)	0.402
Shock	0 (0.0%)	0 (0.0%)	0 (0.0%)	0 (0.0%)	0 (0.0%)	1.000
Syncope	0 (0.0%)	1 (7.1%)	0 (0.0%)	1 (2.6%)	0 (0.0%)	0.558
**ECG at Presentation**						
Normal	2 (13.3%)	6 (42.9%)	0 (0.0%)	8 (21%)	12 (100%)	<0.001
ST elevation	5 (33.3%)	3 (21.4%)	1 (11.1%)	9 (23.6%)	0 (0.0%)	0.048
Diffuse ST elevation	1 (6.67%)	0 (0.0%)	0 (0.0%)	1 (2.6%)	0 (0.0%)	0.558
ST depression	1 (6.67%)	1 (7.1%)	0 (0.0%)	2 (5.3%)	0 (0.0%)	0.402
Negative T waves	2 (13.3%)	2 (14.3%)	3 (33.3%)	7 (18.4%)	0 (0.0%)	0.092
CRP peak (mg/L)	35.30 ± 46.84	1.29 ± 4.81	54.08 ± 106.65	22.82 ± 53.77	5.7± 4.2	<0.001
TnI HS peak ng/L	10,435.93 ± 13,276.89	8023.21 ± 15,864.10	4353.25 ± 5861.74	8446.96 ± 13,515.65	6.3 ± 3.4	<0.001

* All patients vs. healthy controls.

**Table 2:** Echocardiographic and CT parameters.

Echo Parameters						
Variable	Myocarditis (*n* = 15)	MINOCA (*n* = 14)	Takotsubo Cardiomyopathy (*n* = 9)	All Patients (*n* = 38)	Healthy Controls (*n* = 12)	*p*-Value *
Left ventricle EDVI	67.00 ± 12.94	67.14 ± 24.07	62.75 ± 10.34	66.55 ± 17.90	61 ± 9.27	0.467
Left ventricle ejection fraction	56.07 ± 7.89	56.43 ± 11.47	44.50 ± 12.87	54.82 ± 10.56	61.7 ± 5.89	0.133
Regional wall motion	8 (53.3%)	4 (28.6%)	9 (100%)	21 (55.2%)	0 (0%)	<0.001
Pericardial effusion	1 (6.67%)	0 (0.0%)	1 (11.1%)	2 (5.2%)	0 (0%)	0.390
**CT Analysis and pFAI Values**						
Coronary arteries with no lesions	1 (6.7%)	6 (42.9%)	2 (22.2%)	9 (23.7%)	12 (100%)	<0.001
Diameter (mm)	3.32 ± 0.69	3.46 ± 0.91	2.79 ± 0.66	3.26 ± 0.79	3.80 ± 0.88	0.5473
Volume (cm3)	1.20 ± 0.80	1.38 ± 0.65	1.02 ± 0.41	1.23 ± 0.68	1.74 ± 0.56	0.090
Median (HU)	−82.00 ± 13.14	−80.62 ± 14.92	−80.00 ± 13.59	−81.19 ± 13.52	−93.33 ± 7.92	0.041
Mean (HU)	−86.45 ± 11.69	−84.63 ± 13.88	−84.79 ± 13.78	−85.51 ± 12.47	−96.02 ± 6.66	0.0077
Standard deviation (HU)	37.01 ± 5.20	35.28 ± 7.04	36.53 ± 8.47	36.24 ± 6.34	40.02 ± 2.64	0.025

* All patients vs. healthy controls.

**Table 3:** Morphological CMR parameters.

Variable	Myocarditis (*n* = 15)	MINOCA (*n* = 14)	Takotsubo Cardiomyopathy (*n* = 9)	All Patients (*n* = 38)	*p*-Value *	*p*-Value ^†^	*p*-Value ^‡^
EDVi LV (mL/mq)	87.00 ± 13.05	81.69 ± 20.16	77.75 ± 11.56	83.69 ± 16.09	0.410	0.216	0.718
ESVi LV (mL/mq)	36.53 ± 8.23	34.77 ± 12.59	37.00 ± 11.74	35.88 ± 10.30	0.660	0.927	0.758
LV ejection fraction (%)	58.13 ± 6.12	57.92 ± 6.87	53.50 ± 10.63	57.47 ± 6.96	0.933	0.264	0.335
LV mass (g/mq)	64.27 ± 15.40	60.92 ± 14.99	58.75 ± 8.96	62.22 ± 14.36	0.567	0.507	0.790
LV regional wall motion	4 (26.7%)	4 (28.6%)	9 (100%)	17 (44.7%)	0.910	0.0006	0.0010
EDVi RV (mL/mq)	88.13 ± 16.27	80.15 ± 20.05	69.50 ± 6.03	82.56 ± 17.83	0.255	0.003	0.320
ESVi RV (mL/mq)	38.13 ± 11.09	33.85 ± 10.76	28.50 ± 3.70	35.19 ± 10.61	0.310	0.111	0.354
RV ejection fraction (%)	57.33 ± 6.97	58.00 ± 6.03	58.75 ± 8.18	57.78 ± 6.53	0.790	0.731	0.843
RV regional wall motion	1 (6.7%)	0 (0.0%)	0 (0.0%)	1 (2.6%)	0.558	0.558	1.000
**Tissue Characterization**							
LV edema	9 (60.0%)	7 (50.0%)	6 (66.7%)	22 (57.9%)	0.754	0.605	0.484
LV LGE	13 (86.7%)	11 (78.57%)	3 (33.3%)	27 (71%)	0.164	0.0085	0.0005
LGE LV ischemic pattern	0 (0.0%)	11 (78.57%)	0 (0.0%)	11 (29%)	<0.0001	0	<0.0001
RV edema	0 (0.0%)	0 (0.0%)	0 (0.0%)	0 (0.0%)	0	0	0
RV LGE	0 (0.0%)	0 (0.0%)	0 (0.0%)	0 (0.0%)	0	0	0

* *p*-value between myocarditis and MINOCA; ^†^ *p*-value between myocarditis and takotsubo; ^‡^ *p*-value between takotsubo and MINOCA.

**Table 4:** All diseases vs. early detection vs. late detection.

**Variable**	**Myocarditis (*n* = 15)**	**Early CT (*n* = 7)**	**Late CT (*n* = 8)**	**Healthy Controls (*n* = 12)**	*p*-Value *	***p*-Value ^†^**	***p*-Value ^‡^**
Diameter (mm)	3.32 ± 0.69	3.25 ± 0.59	3.38 ± 0.81	3.80 ± 0.88	194	206	363
Volume (cm3)	1.20 ± 0.80	0.92 ± 0.62	1.44 ± 0.90	1.74 ± 0.56	155	32	499
Median (HU)	−82.00 ± 13.14	−80.71 ± 15.86	−83.12 ± 11.26	−93.33 ± 7.92	0.0146	32	28
Mean (HU)	−86.45 ± 11.69	−85.35 ± 14.80	−87.42 ± 9.14	−96.02 ± 6.66	0.0184	0.0437	0.0252
**Variable**	**MINOCA (** ***n*** **= 14)**	**Early CT (** ***n*** **= 9)**	**Late CT (** ***n*** **= 5)**	**Healthy Controls (*n* = 12)**	*p*1-Value *	***p*2-value** ^**†**^	***p*3-Value** ^**‡**^
Diameter (mm)	3.46 ± 0.91	3.36 ± 0.97	3.71 ± 0.79	3.80 ± 0.88	440	372	867
Volume (cm3)	1.38 ± 0.65	1.41 ± 0.75	1.30 ± 0.36	1.74 ± 0.56	261	378	212
Median (HU)	−80.62 ± 14.92	−72.37 ± 10.38	−93.80 ± 11.26	−93.33 ± 7.92	0.0142	0.0001	0.9226
Mean (HU)	−84.63 ± 13.88	−76.91 ± 10.38	−96.99 ± 8.90	−96.02 ± 6.66	0.0160	0.0001	0.8069
**Variable**	**Takotsubo Cardiomyopathy (** ***n*** **= 9)**	**Early CT (** ***n*** **= 4)**	**Late CT (** ***n*** **= 5)**	**Healthy Controls (** ***n*** **= 12)**	***p*** -Value *	***p*** **-Value** ^**†**^	***p*** **-Value** ^**‡**^
Diameter (mm)	2.79 ± 0.66	2.78 ± 1.53	2.95 ± 0.45	3.80 ± 0.88	99	255	267
Volume (cm3)	1.02 ± 0.41	1.04 ± 0.47	0.94 ± 0.28	1.74 ± 0.56	52	59	119
Median (HU)	−80.00 ± 13.59	−77.50 ± 16.26	−82.50 ± 11.26	−93.33 ± 7.92	0.0107	0.0185	0.0378
Mean (HU)	−84.79 ± 13.78	−83.54 ± 16.21	−86.02 ± 12.34	−96.02 ± 6.66	28	40	0.0442

* All diseases vs. healthy controls; ^†^ early CT vs. healthy controls; ^‡^ late CT vs. healthy controls.

**Table 5:** Follow-up data.

Variable	Myocarditis (*n* = 15)	MINOCA (*n* = 14)	Takotsubo Cardiomyopathy (*n* = 9)	All Patients (*n* = 38)	Healthy Controls (*n* = 12)	*p*-Value *
Follow-up	15 (100%)	14 (100%)	9 (100%)	38 (100%)	12 (100.0%)	1.000
ER admissions	8 (53.3%)	8 (57.1%)	0 (0.0%)	16 (42.1%)	0 (0.0%)	<0.001
In-hospital admissions	4	4	0	8	0	<0.001
Chest pain	3 (20.0%)	2 (14.2%)	0 (0.0%)	5 (13.1%)	0 (0.0%)	0.167
ACS	0 (0.0%)	0 (0.0%)	0 (0.0%)	0 (0.0%)	0 (0.0%)	1.000
Arrhythmias	1 (6.67%)	2 (7.1%)	0 (0.0%)	2 (5.3%)	0 (0.0%)	0.402
Syncope	0 (0.0%)	0 (0.0%)	0 (0.0%)	0 (0.0%)	0 (0.0%)	1.000
Death	0 (0.0%)	0 (0.0%)	0 (0.0%)	0 (0.0%)	0 (0.0%)	1.000

* All patients vs. healthy controls.

## Data Availability

The data that support the findings of this study are available on request from the corresponding author (V.P.).
